# Supraglottic Kaposi's Sarcoma in HIV-Negative Patients: Case Report and Literature Review

**DOI:** 10.1155/2016/1818304

**Published:** 2016-06-08

**Authors:** Ela A. Server, Yusuf M. Durna, Ozgur Yigit, Erol R. Bozkurt

**Affiliations:** ^1^Department of Otolaryngology, Istanbul Research and Training Hospital, 34540 Istanbul, Turkey; ^2^Department of Pathology, Istanbul Research and Training Hospital, 34540 Istanbul, Turkey

## Abstract

This paper presents a case report of an HIV-negative, supraglottic Kaposi's sarcoma patient. The 80-year-old male patient was admitted with complaints of hoarseness, difficulty in swallowing, and a stinging sensation in his throat for approximately six months. The endoscopic larynx examination revealed a lesion which had completely infiltrated the epiglottis, reached right aryepiglottic fold, was vegetating, pink and purple in color, multilobular, fragile, and shaped like a bunch of grapes, and partially blocked the bleeding airway passage. The case was discussed by the hospital's head-neck cancer committee and a surgery decision was made. A tracheotomy was performed under local anesthesia before the operation due to respiratory distress and endotracheal intubation difficulty. Direct laryngoscopy showed that the mass was limited in the supraglottic area, had invaded the entire left aryepiglottic fold and one-third of the front right aryepiglottic fold, and completely covered epiglottis. It should be remembered that although rare, Kaposi's sarcoma may be encountered in larynx malignancy cases. Disease-free survival may be achieved through local excision and postoperative radiotherapy.

## 1. Introduction

Kaposi's sarcoma is a vascular tumor that was first described by Kaposi in 1872 [1] and has a low potential of malignancy. Kaposi's sarcoma is caused by human herpesvirus 8 (HHV-8) and often seen in Human Immunodeficiency Virus- (HIV-) infected patients [2, 3]. Its multifocal localization areas have been defined. It is frequently found in the lower extremities, facial skin, and genital and oropharyngeal mucosa but may also appear in gastrointestinal and respiratory tract mucosa, in addition to other less common areas, such as the larynx. This uncommon location is usually related to HIV [4, 5]. This paper presents a case report and literature review of a rare case of supraglottic, epiglottis, and aryepiglottic fold-derived, HIV-negative, HHV-8 positive Kaposi's sarcoma.

## 2. Case Report

**Figure 1:**
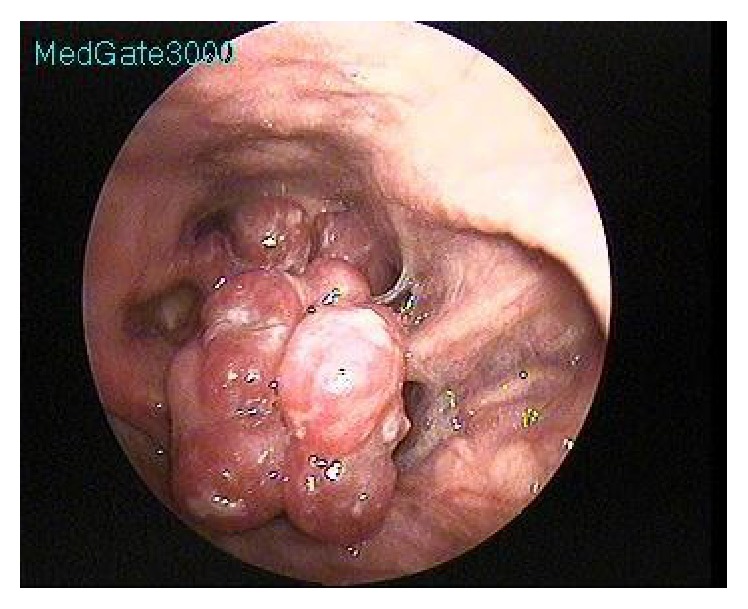
Pink and purple colored mass with lobular contours on the epiglottis laryngeal surface near the aryepiglottic fold.

The 80-year-old male patient complained of hoarseness and a stinging sensation in his throat for approximately six months and was then transferred to our clinic. The endoscopic larynx examination revealed a lesion that completely infiltrated the epiglottis and reached the right aryepiglottic fold. It was vegetating, pink and purple in color, multilobular, fragile, and shaped like a bunch of grapes and was covering the bleeding airway passage (Figure 1).

Before arriving at our clinic, a punch biopsy was done and suggested a diagnosis of Kaposi's sarcoma. The patient reported that he used to smoke approximately fifty packs of cigarettes per year but had not smoked in the last five years. He had no history of systemic disease or infection. Nothing was remarkable in routine blood analyses. ELISA tests for HIV-1, HBV, and HCV were negative. A positron emission tomography (PET-CT) showed that no other areas were affected. The case was discussed by our hospital's head-neck cancer committee and a surgery decision was made. A tracheotomy was performed under local anesthesia before the operation due to respiratory distress and endotracheal intubation difficulty. The direct laryngoscopy showed that the mass was limited in the supraglottic area, had invaded the entire left aryepiglottic fold and one-third of the front right aryepiglottic fold, and completely covered epiglottis. The laryngeal ventricle, vallecula, arytenoids, interarytenoid area, and glottic and subglottic areas were intact. The mass was found to be bleeding and fragile.

**Figure 2:**
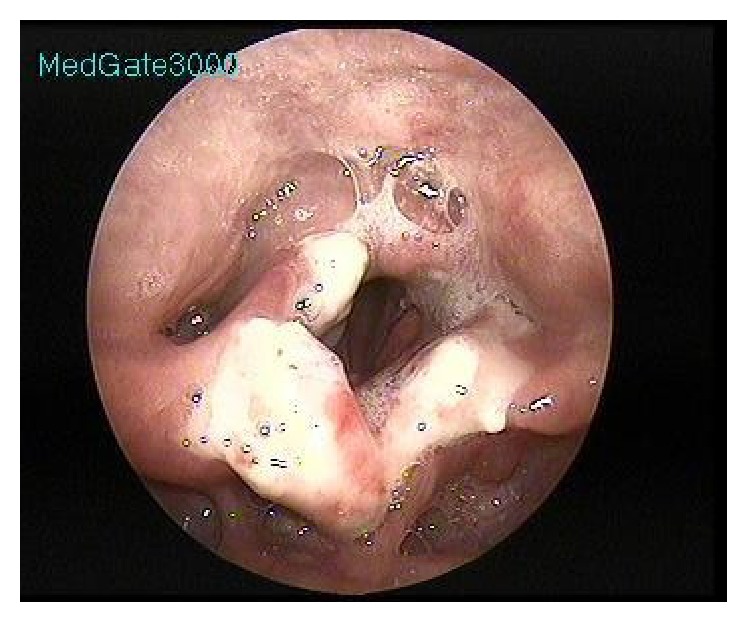
One week after endolaryngeal laser surgery, glottic levels were intact and white-colored granulation tissues were observed.

An epiglottectomy was conducted with a diode laser and partial resection was performed on the aryepiglottic folds (Figure 2).

**Figure 3:**
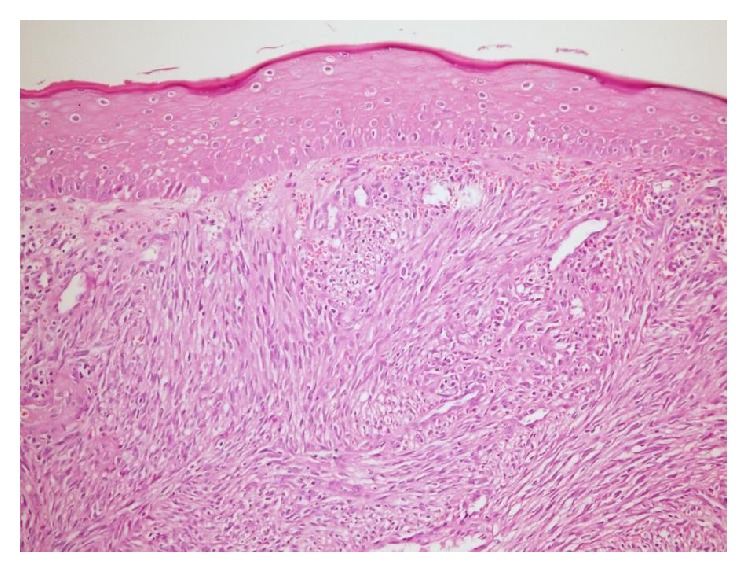
Tumoral mass consisting of fusiform cells under the surface epithelium (original magnification, H&E ×40).

**Figure 4:**
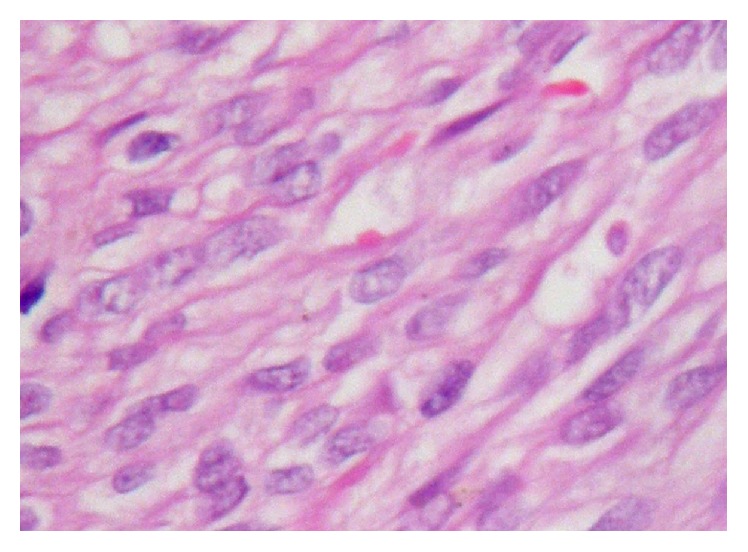
Some fusiform cells creating slits and bundle structures involving erythrocytes (original magnification, H&E ×100).

**Figure 5:**
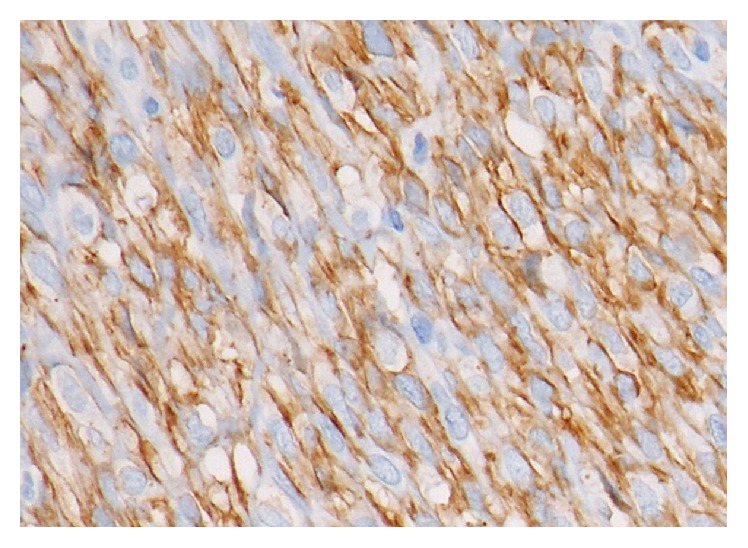
Common CD31 positivity in tumoral cells (original magnification, H&E ×100).

**Figure 6:**
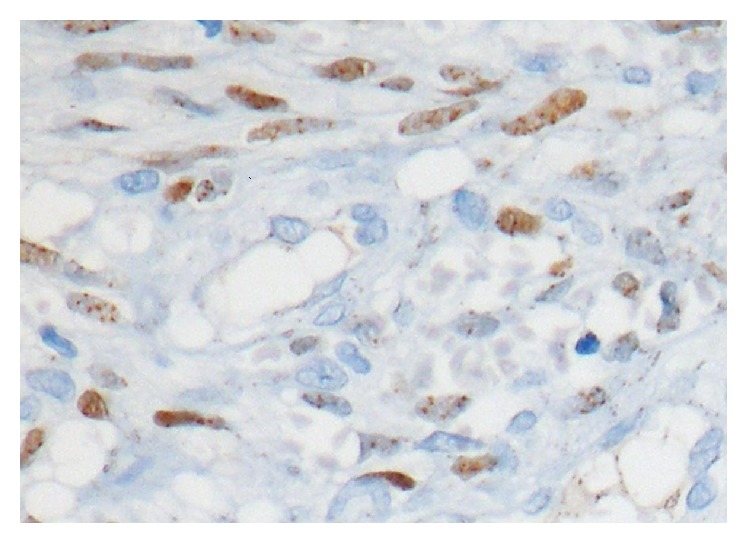
Apparent nuclear HHV-8 positivity in tumor cells (original magnification, H&E ×100).

In the histopathological examination of the mass, a tumoral mass consisting of fusiform cells under the surface epithelium was observed in preparations stained with hematoxylin-eosin (Figure 3). Some of the fusiform cells constituted slits and bundle structures involving erythrocytes (Figure 4). After staining the tumoral tissue with common CD31, the diagnosis of Kaposi's sarcoma was made and an apparent nuclear HHV-8 positivity was observed (Figures 5 and 6). When the pathological piece was analyzed, surgical borders were found to be positive. The patient was referred to the radiation oncology department for radiotherapy treatment (RT). The patient was disease-free after one year of follow-up and 33 RT sessions.

## 3. Discussion

Kaposi's sarcoma, which is a vascular tumor, has four described types: common, endemic, immunosuppression or transplantation-related, and epidemic or AIDS-related. The most aggressive form is epidemic, observed in HIV-positive patients [6]. Iatrogenic tumors are seen in immunosuppressive treatment areas and do not progress aggressively. Endemic KS was described in Sub-Saharan indigenous Africans prior to the AIDS epidemic. The average age of incidence is 35–39 among men and 22–39 among women [7]. Common tumors are widespread in Mediterranean countries such as Italy, Greece, and Israel in people over 70 years old. Clinically, it does not progress aggressively and does not lead to death. A study conducted in Greece found that it was more common among men than women between 1979 and 1983 [8]. This paper describes a case of common Kaposi's sarcoma, as the patient was HIV-negative.

HIV-1, HHV-8, CMV, mycoplasma, and Chlamydia infections have been associated with the etiology of common Kaposi's sarcoma. It is generally observed in extremities and rarely in oral cavities, as a cutaneous, red to purple lesion. The majority of patients reported in the literature are AIDS or immunosuppressed patients; HIV-negative and nonimmunosuppressed cases are rare [9–11].

Six Kaposi's sarcoma cases were reported in 1996 by Friedman et al., which involved the larynx [12]. In 1997, Schiff et al. published two epiglottis-related cases, one HIV-negative and one HIV-positive [13]. In a review, Patrikidou reported that HIV-negative Kaposi's sarcoma was localized on larynx at a rate of 5% [12]. Our patient was HIV-negative and HHV-8 positive, and the tumor had supraglottic localization.

Aphonia, dysphagia, hoarseness, and stridor are symptoms that may arise in tumors on the larynx. Red or purple mass lesions may be observed in the endoscopic larynx examination [13]. Since it is a vascular tumor, the risk of bleeding should not be forgotten in the biopsy. The histopathology of Kaposi's sarcoma shows that slit-like vascular cavitations are surrounded by fusiform cells. Fusiform cells are stained positive with CD34, Factor 8, and CD31 [14]. In our case report, positive CD31 and HHV-8 indicated Kaposi's sarcoma.

Treatment for HIV-negative cases involves local excision, cryotherapy, radiotherapy, vinblastine injection into lesions, and systemic single-agent or combination chemotherapy [13]. Serious airway obstruction may develop in larynx-localized Kaposi's sarcoma cases and a tracheotomy may be required. Radiotherapy may be offered after local excision as it is a radiosensitive tumor. In our case, radiotherapy was started after the laser epiglottectomy performed under general anesthesia. Patients should be monitored closely during follow-up since systemic spread and local recurrence may develop.

## 4. Conclusion

Although rare, common Kaposi's sarcoma may be encountered on the larynx. Disease-free survival may be achieved through local excision and postoperative radiotherapy.
